# The ethology of wolves foraging on freshwater fish in a boreal ecosystem

**DOI:** 10.1098/rsos.230210

**Published:** 2023-05-24

**Authors:** Danielle R. Freund, Thomas D. Gable, Sean M. Johnson-Bice, Austin T. Homkes, Steve K. Windels, Joseph K. Bump

**Affiliations:** ^1^ Department of Fisheries, Wildlife, and Conservation Biology, University of Minnesota, 2003 Upper Buford Circle, St Paul, MN 55108, USA; ^2^ Department of Biological Sciences, University of Manitoba, 50 Sifton Road, Winnipeg, Manitoba, Canada R3T 2N2; ^3^ Voyageurs National Park, National Park Service, 360 Highway 11 East, International Falls, 56649 MN, USA

**Keywords:** *Canis lupus*, wolf predation, ambush hunting, predator prey relations, fish spawning, boreal forest

## Abstract

Through global positioning system (GPS) collar locations, remote cameras, field observations and the first wild wolf to be GPS-collared with a camera collar, we describe when, where and how wolves fish in a freshwater ecosystem. From 2017 to 2021, we recorded more than 10 wolves (*Canis lupus*) hunting fish during the spring spawning season in northern Minnesota, USA. Wolves ambushed fish in creeks at night when spawning fish were abundant, available and vulnerable in shallow waters. We observed wolves specifically targeting sections of rivers below beaver (*Castor canadensis*) dams, suggesting that beavers may indirectly facilitate wolf fishing behaviour. Wolves also cached fish on shorelines. We documented these findings across five different social groups at four distinct waterways, suggesting that wolf fishing behaviour may be widespread in similar ecosystems but has probably remained difficult to study given its annual brevity. Spawning fish may serve as a valuable pulsed resource for packs because the spring spawning season coincides with low primary prey (deer *Odocoileus virginianus)* availability and abundance, and when packs have higher energetic demands owing to newly born pups. We demonstrate the flexibility and adaptability of wolf hunting and foraging behaviour, and provide insight into how wolves can survive in a myriad of ecosystems.

## 1. Introduction

How species respond and adapt to varying environmental conditions is often dependent on diet and dietary flexibility [1–4]. Generalist predators can exhibit high dietary plasticity and switch to alternate prey or food sources when they become available [5,6]. This plasticity is particularly important for predators capturing prey that are only available or vulnerable for short periods of time (e.g. neonatal prey, [7]). Studying the dietary plasticity of large ambush predators is particularly difficult owing to their elusiveness and large home ranges [1,8].

Grey wolves (*Canis lupus*) are generalist predators that can respond to and capitalize on newly available food within days. Wolves are adept at exploiting short-term, seasonal food pulses such as geese (*Branta* spp*.*) and molting birds [9], carcasses of livestock or hunter-killed prey [10,11], berries [12] and salmon (*Oncorhynchus* spp*.*) [13]. This dietary plasticity enables wolf occupation of a wide range of biomes (e.g. deserts, tundra, forests, plains) across the northern hemisphere [14]. Wolf responses to changing food availability and their use of alternative prey can have implications for prey population dynamics in the various systems wolves occupy [1].

Over the past two decades, a number of studies have recorded wolves consuming salmon and responding to the pulsed resource of the salmon spawning season in Alaska and British Columbia [13,15–20]. In coastal British Columbia, wolves target salmon regardless of the availability of their primary prey (deer), indicating that salmon are an important seasonally available food source for wolves even when primary prey are abundant. Hunting salmon is less risky (i.e. smaller chance of injury from prey, shorter travelling and handling time) for wolves compared to hunting and killing ungulates. Salmon also have enhanced fat and energy value over deer [18] and can provide a food resource equal in magnitude to ungulates [15]. The fact that salmon are a more easily acquirable prey that wolves hunt independent of ungulate abundance suggests that wolves may prey on salmon wherever wolves and salmon geographically overlap [16]. For example, in northern Québec and Labrador, Canada, wolves feed on Arctic char (*Salvelinus alpinus—*a member of the Salmonidae family) in shallow rivers during the late-summer and early autumn spawning period [1].

Despite these findings, wolf use of fish, particularly non-salmonid species, is still poorly understood. A large portion of available literature on wolf consumption of fish includes stable isotope, scat and stomach content analyses [1,13,15,17–24]. These studies are valuable to quantify the relative amount of fish wolves consume compared to other foods, but investigators have paid considerably less attention to how wolves acquire fish when they are available [16,25,26]. Previous accounts of wolf consumption of lake trout (*Salvelinus namaycush*) and northern pike (*Esox lucius*) [22,23] indicate that wolves consume freshwater fishes, but until recently it remained unclear whether wolves scavenge or actively hunt freshwater fish species [22–24].

In 2017 Gable *et al*. [27] documented two global positioning system (GPS)-collared wolves (a yearling and breeding male) hunting and killing white suckers (*Catostomas commersoni*) at a creek in northern Minnesota, USA during the spring spawning season. Gable *et al*. [27] directly observed the yearling wolf hunting and consuming fish, and GPS-collar data provided evidence that the breeding male (father of the yearling) also hunted and consumed fish. This was a novel record in wolf ecology because it confirmed wolves hunt freshwater fishes. However, these observations provide no indication if wolf fishing behaviour is common in different social groups or recurs across years.

Since the behavioural observation in 2017, we documented several wolves from different social groups hunting and killing fish in the Greater Voyageurs Ecosystem (GVE), Minnesota, USA using GPS-collar locations, remote cameras, GPS collars equipped with cameras, and field observations. Together, these lines of evidence produce detailed observations of wolves fishing in a freshwater ecosystem. Our objective is to provide an in-depth description and summary of this elusive behaviour, including (i) when wolves hunt fish, (ii) where wolves primarily fish, and (iii) the dominant hunting strategies used to catch fish. Our work provides evidence that wolf ‘fishing’ behaviour is probably widespread across similar boreal ecosystems but has remained hard to study given its ephemeral nature.

## 2. Material and methods

### 2.1. Study area: the Greater Voyageurs Ecosystem

**Figure 1.**
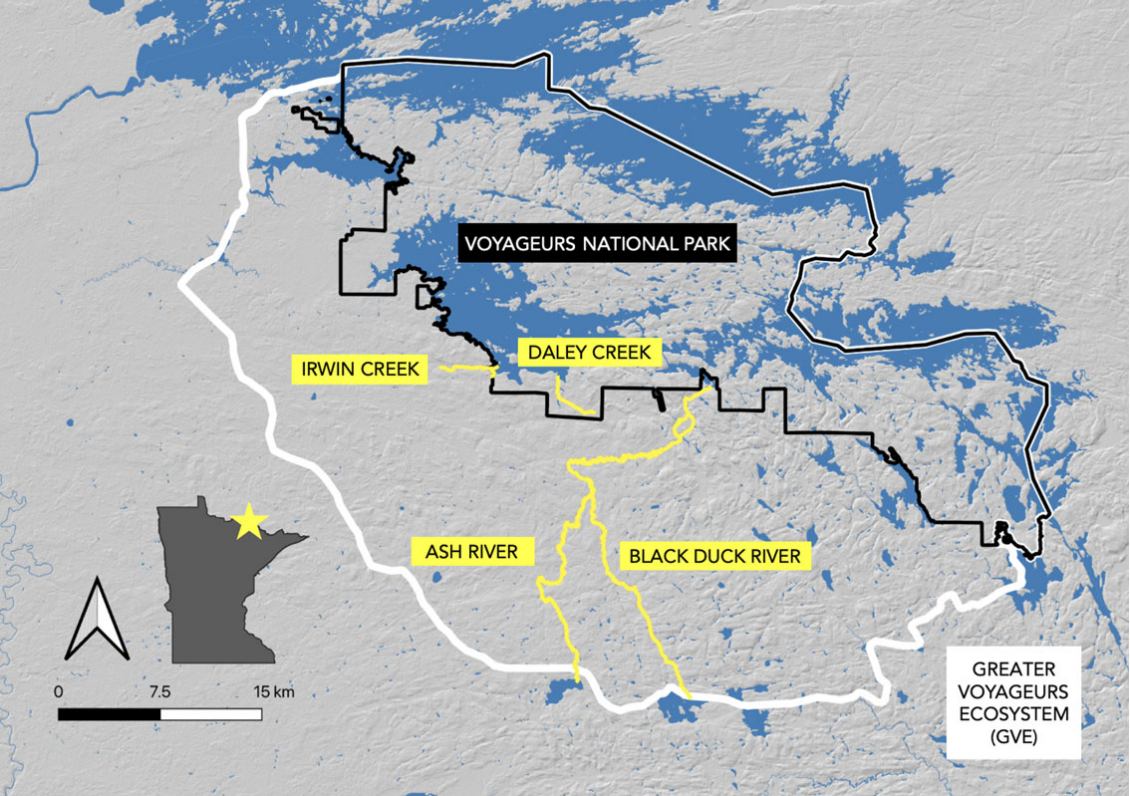
Map of the Greater Voyageurs Ecosystem (GVE; white line). Map includes Voyageurs National Park (black line), and creeks/rivers (yellow lines) where wolves were recorded fishing in northern Minnesota, USA.

The GVE (figure 1) is made up of Voyageurs National Park in the north, and US Forest Service, state-owned and commercial forest in the southern and central portions [12]. The GVE contains numerous creeks and tributaries that are connected to four large lakes (Kabetogama, Rainy, Namakan and Sand Point). Typically, lakes freeze during late October to mid-November and most waterways (e.g. creeks, rivers) are ice-free by mid-April [28].

Wolf packs occupy the entirety of the GVE and the area has maintained high wolf densities for greater than 30 years [29,30]. Wolf densities from 2017–2021 were estimated to fluctuate between approximately 45 and approximately 75 wolves 1000 km^2^ [31]. White-tailed deer (*Odocoileus virginianus*) are the primary annual prey of wolves (density = 2–4 deer km^2^), with adult deer making up approximately 40% of wolf diet and fawns making up approximately 20% of wolf diet from April to October [11,32,33]. Beavers (*Castor canadensis*) are important seasonal prey for wolves because of their abundance (density = approx. 1 colony km^2^) [11], constituting up to 42% of wolf pack diets during the ice-free season (April to October) when they are vulnerable to predation [34]. Moose (*Alces americanus*) are relatively rare in the GVE with only 0.15 moose km^2^ in Voyageurs National Park, and are rarely detected in wolf diet [11,35]. More than 50 fish species, including white suckers (*Catostomus commersonii*), yellow perch (*Perca flavescens*), northern pike (*Esox lucius*), and blacknose shiner (*Otropis heterolepis*) inhabit the lakes of the GVE and use the connected waterways (e.g. streams, creeks) as spawning habitat in the spring [28]. From 2012 through to 2015 fish comprised a negligible proportion of diets for wolves in and adjacent to Voyageurs National Park [27,32,36].

### 2.2. Documenting wolf fishing behaviour

We documented fishing behaviour every summer in the GVE from 2017 to 2021 through four different sources of data: (i) a direct observation of a wolf fishing, (ii) wolves fitted with GPS collars [27], (iii) remote camera footage, and (iv) a GPS collar with a camera attached that recorded videos from the wolf's perspective. All data were assessed as an amalgamation across years, data types, and wolf packs.

We ear-tagged and fitted wolves with GPS-collars via the methods outlined in Gable *et al*. [29] following Institutional Animal Care and Use Committee approvals by the U.S. National Park Service (MWR VOYA WINDELS WOLF) and the University of Minnesota (protocol ID: 1905-37051A). Ear tags provided unique IDs for each wolf that we used to recognize wolves in remote camera videos. Wolves were GPS-collared as part of the annual data collection of the Voyageurs Wolf Project (VWP), a larger research project studying the predation behaviour and reproductive ecology of wolves in the GVE.

#### 2.2.1. In-field observations: incidentally recording fishing behaviour

In 2017, a yearling from the Bowman Bay Pack (V046) was incidentally observed hunting fish along a shallow portion of Irwin Creek (see [27]). Gable *et al*. [27] examined the time spent fishing by the yearling wolf and their pack mate (V034) that year via 20 min and 12 h GPS-collar locations respectively. Despite the difference in fix-interval rate, both wolves showed similar spatio-temporal patterns in fishing behaviour and the estimated time each wolf spent ‘fishing’ only differed by 4%. The time these two wolves spent fishing as determined by Gable *et al*. [27] was used in our analysis.

#### 2.2.2. GPS collar data: GPS clusters

After the initial observations of wolves fishing in 2017 [27], to determine if and when subsequent wolves fished in the GVE, we searched GPS clusters of wolves fitted with 20 min fix interval collars. We considered a cluster as two or more consecutive GPS locations within a 200 m radius of the first location of the cluster [27,29]. When at clusters, we searched a 20 m radius around all GPS locations associated with the cluster to identify prey remains and other evidence of predation events. We assumed that wolves hunted and killed fishes at GPS clusters where fresh fish scales, gill plates, blood and intestinal contents, fins and/or fish bones were found. Notably, we only considered wolves to have hunted fishes at sites where wolf sign was abundant (i.e. wolf hair, scats and tracks) and there was no evidence of other predators (scats, tracks or hair from bears (*Ursus amercanus*), raccoons (*Procyon lotor*), otters (*Lontra canadensis*) etc.). If fish remains were old (tissue was hard and black, bones were bleached by the sun, etc.) we did not assume the wolf actively fished because they may have been scavenging. We did not estimate the number of fish killed by wolves at clusters owing to the large amounts of fish remains present at each cluster site [27]. When possible, we identified the species of fish from the remains at the clusters. When we found evidence that wolves were fishing, we noted the depth of the water (less than 1 or greater than 1 m deep) and whether there were any active beaver dams nearby.

#### 2.2.3. Remote cameras: capturing fishing behaviour

In 2018 and 2019, we placed remote cameras along Irwin Creek to capture photographs and video footage of the Bowman Bay pack fishing (figure 1), the only pack we have remote camera data for. We only placed remote cameras along Irwin Creek because Irwin Creek is where we observed wolves fishing in the field in 2017, and available resources only permitted cameras at Irwin Creek. In 2018, we placed one Reconyx Silent Image (set to take bursts of five photos 1 s apart when triggered) and one Reconyx Ultrafire (set to take 30 s videos with a 1 s delay between videos) along the creek edge from 7 May to 23 May. In 2019, we placed 21 remote cameras (all Browning Spec Ops Advantage) along Irwin Creek from 19 April to 17 May and programmed the cameras to record 20 s videos when triggered with a 1 s delay between videos.

We considered an ‘event’ as a series of photos or videos of the same wolf(ves) where 10 min or less elapsed between sequential photos or videos. If greater than 10 min elapsed between sequential pictures or videos, then we considered it a unique event (similar to the image classification in [37,38]). We recorded the date, start and end time, duration, number of wolves, if the individuals were collared and their ID, and the primary behaviour of the wolf(ves) during the event. We classified observed behaviours into seven categories: *on creek bank*, *wading, successful fishing attempt, failed fishing attempt, carrying fish, caching fish and unknown fishing attempt* (table 1; electronic supplementary material, video S1). Owing to a camera malfunction, dates were not recorded for 11 events captured between 19 April 2019 and 25 May 2019. Behaviours captured in these events were analysed, but videos from these cameras were not used to define the start and end dates of fishing periods (see ‘Categorizing wolf fishing period’ section below).

**Table 1.** Ethogram of wolf fishing behaviours recorded along Irwin Creek in the Greater Voyageurs Ecosystem via remote cameras in 2018 and 2019.

specific behaviour	definition
on creek bank	wolf runs, walks or stands on the bank of creek
wading	wolf walks or runs in the water of the creek
complete fishing attempt	wolf is observed successfully ambushing and catching a fish but is not observed caching the catch
failed fishing attempt	wolf unsuccessfully attempts to catch a fish that is visible
carrying fish	no observation of wolf catching a fish, but the wolf carries a fish in its mouth without dropping and leaving it (i.e. the wolf has the fish in its mouth for the entire duration of the video)
caching fish	wolf has a fish in its mouth, drops the fish on the creek bank, and leaves the fish (i.e. the wolf does not eat the fish but rather caches it)
unknown fishing attempt	wolf attempts to catch a fish (lunges towards water, plunges snout in water, or chases after visible fish in frame) but the outcome of the attempt is not visible in the video footage (i.e. wolf leaves video frame, video cuts out etc.)

#### 2.2.4. Camera collar: capturing fishing behaviour

In 2020, a lone male wolf was fitted with the first 20 min fix interval collar with a built-in video camera to be deployed on a wild wolf (Vectronic-Aerospace Vertex Plus Collar). The collar recorded one 30 s video at the beginning of every hour during daylight hours (approximately 6.00–20.00) for 43 days from 28 April 2020 to 9 June 2020 before the collar dropped off as programmed. We then examined all video footage and recorded the behaviours observed in each video (running, walking, bedding, licking paws, eating, sniffing, etc.; table 2; electronic supplementary material, video S2). We also recorded the species of any identifiable prey that the wolf consumed.

**Table 2.** Summary of wolves observed fishing within the Greater Voyageurs Ecosystem, Minnesota between 2017 and 2021. (The period each tagged wolf presumably spent hunting and handling fish (referred to as the ‘fishing period’) was designated for an individual wolf from the date it was first observed fishing via searched GPS clusters, GPS locations, or remote camera footage to the last date such observations were made. The fishing periods for V034 and V046 in 2017 were defined from Gable *et al.*, [27]. Uncollared wolves include wolves without collars, and wolves we were not able to identify as collared or not (head was out of frame of video from remote camera etc.). V034 (2017 and 2019) and V067 (2018 and 2019) were observed fishing in two years. V067 was observed in one video wading in Irwin Creek via remote cameras in 2018.)

year	wolf ID	pack	social status	sex	collar fix interval	river / creek	fishing period	length of fishing period (days)	fishing period defined by
2017	V034	Bowman Bay	breeding	male	12 h	Irwin Creek	17 April–17 May	31	Gable *et al*. [27]^a^
	V046	Bowman Bay	subordinate	male	20 min	Irwin Creek	21 April–18 May	28	Gable *et al*. [27]^a^
2018	V060	Bowman Bay	subordinate	female	12 h	Irwin Creek	12 May–18 May	7	remote camera
	V062	Bowman Bay	subordinate	male	20 min	Irwin Creek	9 May–25 May	17	clusters
	V067	Bowman Bay	subordinate	female	collar failed	Irwin Creek	n.a.	n.a.	n.a.
	uncollared	unknown	unknown	unknown	n.a.	Irwin Creek	7 May–16 May	10	remote camera
2019	V034	Bowman Bay	breeding	male	n.a.	Irwin Creek	4 May–21 May	18	remote camera
	uncollared	unknown	unknown	unknown	n.a.	Irwin Creek	29 April–21 May	23	remote camera
2020	V077	Paradise	breeding	male	20 min	Ash River	19 May–29 May	11	clusters
	V089	n.a. (Lone Wolf)	lone	male	20 min	Ash River	20 May–28 May	9	camera collar
2021	V071	Lightfoot	breeding	male	20 min	Daley Creek	24 April–17 May	24	clusters
	V094	Half-Moon	breeding	male	20 min	Irwin Creek	2 May–9 May	8	clusters
	O0C	Windsong	subordinate	male	20 min	Ash River & Black Duck	26 May–7 July	43	clusters

^a^See reference [27].

### 2.3. Categorizing wolf fishing period

We refer to the time that each collared wolf spent hunting and handling fish as the ‘fishing period’, defined as the first and last date each wolf was documented fishing based on the four methods described above [27]. If GPS cluster data were not available, we used remote camera or camera collar footage of wolves fishing to define fishing periods (table 2). In remote camera footage, we could not reliably identify individuals without collars. Consequently, uncollared wolves captured on remote cameras, or wolves where we were unable to detect a collar, were classified as one group within the year we recorded them (table 2).

To examine how the spatial movements of wolves change in response to the availability of fish, we examined the time individual wolves spent at and around specific ‘fishing water sources' before, during, and after wolves hunted and handled fish [27]. We defined ‘fishing water sources’ as any river or creek where in field observations, clusters, remote camera evidence and/or camera collar footage indicated wolves fished. We quantified the amount of time wolves spent fishing and handling fish at fishing water sources as per Gable *et al*. [27]. Specifically, we quantified the amount of time individual wolves spent within 20 m and 500 m of fishing water sources during an individual fishing period from 20 min GPS fixes. We considered time spent within 500 m of a fishing water source as an index of the time wolves spent hunting and handling fish [27]. Our cluster data and camera collar footage indicate that wolves in the GVE commonly bed down 500 m from fishing water sources to consume fish between fishing events. We considered time spent within 20 m of a fishing water source as an index of the time wolves spent actively hunting fish. This method follows previous studies that have quantified wolf handling time of prey [39]. Specifically, Tallian *et al*., [39] quantified the time wolves spent consuming prey as the GPS locations within 100 or 200 m of a carcass site, and the time wolves spent handling prey as the GPS locations within 100 or 200 m of a carcass site as well as all GPS locations where the first point within a GPS cluster occurred within 12 h of a kill site. We created histograms of the locations recorded within the buffers on an annual and daily scale, and visually examined the peaks of those histograms (similar to [40]). Spatial analysis was completed via QGIS (v. 6.4.6 Greenbelt) and R v. 4.0.4 (2021-02-15).

Our attempt to define when wolves fished is an index and probably does not capture the full extent of the behaviour because both remote camera footage and 12 h GPS locations might be too infrequent to reliably record short-term behaviours such as fishing. Cameras provide an incomplete record of fishing behaviour because they do not exhaustively cover areas where wolves are fishing, resulting in missed fishing behaviours. Additionally, wolves probably were not exclusively hunting, consuming, and handling fish within 20 and 500 m of fishing water sources where we observed them fishing during their fishing period. Each data source only opportunistically captured a particular aspect of the fishing behaviour and in some instances, from only one or a few individuals. However, by synthesizing these varied and diverse lines of evidence, we have been able to provide deeper insight into this unique behaviour.

## 3. Results

We documented 10 collared wolves from five different social groups fishing at four different rivers or creeks between 2017 and 2021 in the GVE (table 2 and figure 1). The 10 fishing wolves included eight males and two females, of which four were breeding individuals, five subordinates and one lone wolf. Five collared wolves were from the same pack (Bowman Bay), whereas the other four collared wolves all belonged to different packs. These four wolves from different packs were the only individuals within their respective packs that were fitted with GPS collars (table 2). The 10 fishing wolves made up approximately 34% of the total wolves collared and studied by VWP during the spring spawning season from 2017 to 2021 (10 out of 29 total collared wolves). We did not have sufficient information to determine if several collared individuals fished (e.g. wolves collared after the spring spawning season, or wolves with collars that failed). In all observations of wolves successfully killing and consuming fish, we recorded wolves killing white suckers.

We recorded GPS locations for nine of the 10 fishing wolves, as one collar failed immediately after deployment (V067). However, remote cameras recorded V067 fishing a year after she was collared. Seven of the nine successful GPS collars recorded wolf locations every 20 min and two recorded locations every 12 h. We searched clusters from six of the seven wolves with 20-min fix location collars to identify fishing sites (V046, V062, V077, V071, V094 and O0C; table 2, see the Camera Collar Data section below for information on the seventh wolf, V089, with a 20 min fix interval collar). Gable *et al*. [27] searched a subset of wolf V046's clusters until her collar dropped off in late summer.

**Figure 2.**
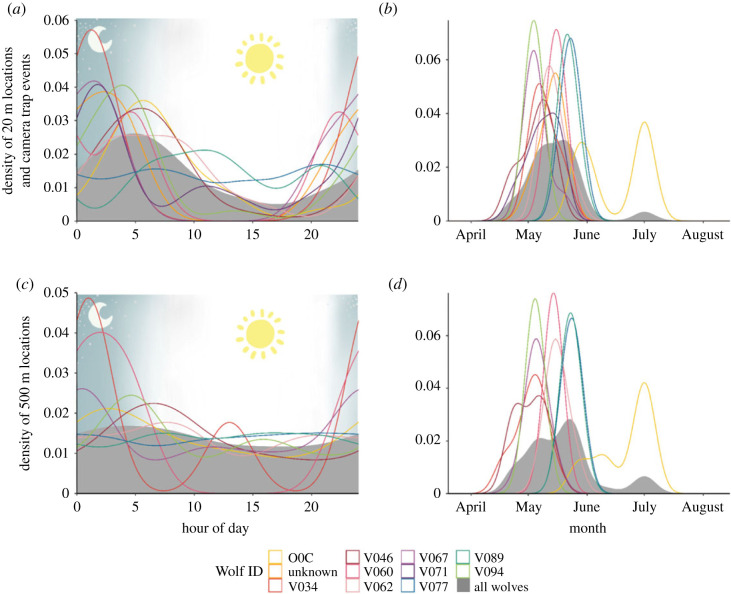
Hourly (*a*,*c*) and seasonal (*b*,*d*) patterns of wolf fishing behaviour in the Greater Voyageurs Ecosystem, Minnesota during 2017–2021. Using GPS collars and remote cameras, we quantified when wolves spent time within 20 (*a*,*b*) and 500 m (*c*,*d*) of creeks/rivers where they caught fish. Locations within 20 m of creeks/rivers correspond to when wolves actively hunted fish (i.e. does not include handling time), whereas locations within 500 m probably include time to hunt and then consume fish (Gable *et al*. [27]). Remote camera data was only included in 20 m graphs (*a*,*b*) because remote cameras were placed on the creek/river banks (e.g. within 20 m). Individual coloured lines indicate individual wolves.

**Figure 3.**
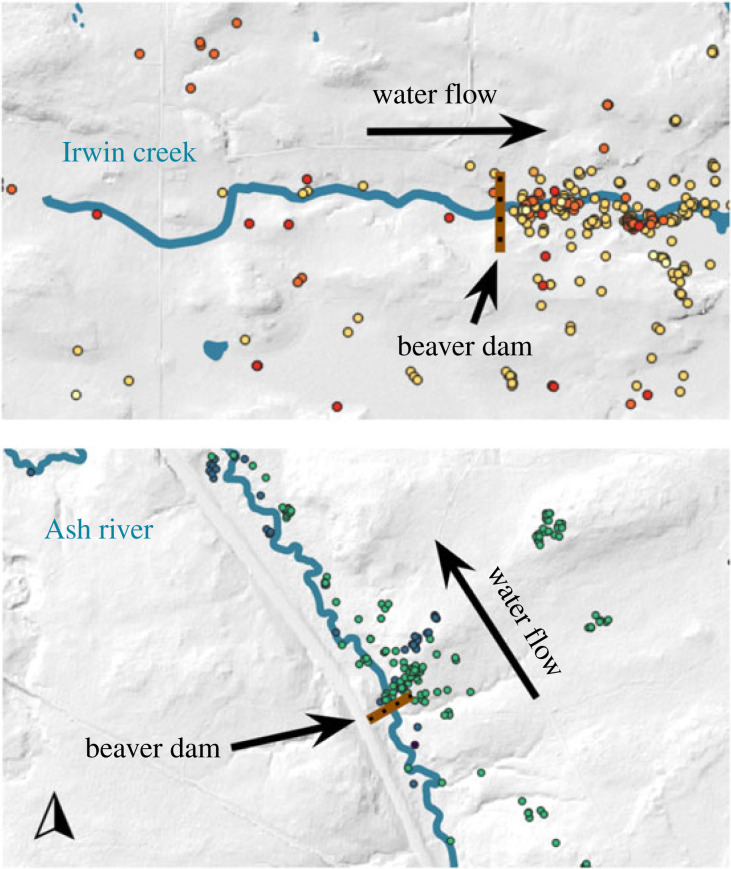
GPS locations of wolves documented fishing downstream of beaver dams in northern Minnesota. Different colours indicate different collared individuals: five wolves at Irwin Creek in 2017, 2018 and 2020, and three wolves at Ash River in 2020 and 2021. GPS locations depicted are during each wolf's individual ‘fishing period’.

Each wolf that hunted fish did so during the spring spawning season between April and May regardless of the year (table 2 and figure 2). We refer to the time that each collared wolf spent hunting and handling fish as the ‘fishing period’ [27]. Only one wolf hunted and killed fish in June and July (wolf O0C). Wolf fishing periods lasted on average 19 ± 10.5 (s.d.) days with a maximum fishing period length of 43 days by wolf O0C. Wolf O0C had two distinct peaks in fishing behaviour in June and July, indicating that he was not intensely fishing for all 43 days of his fishing period (figure 2). For example, O0C only spent time within 500 m of the water source where he fished for 27 of those 43 days. Wolves mainly fished between the hours of 20.00 and 6.00 (figure 2) in shallow waterways less than 1 m deep downstream of beaver dams (figure 3).

**Figure 4.**
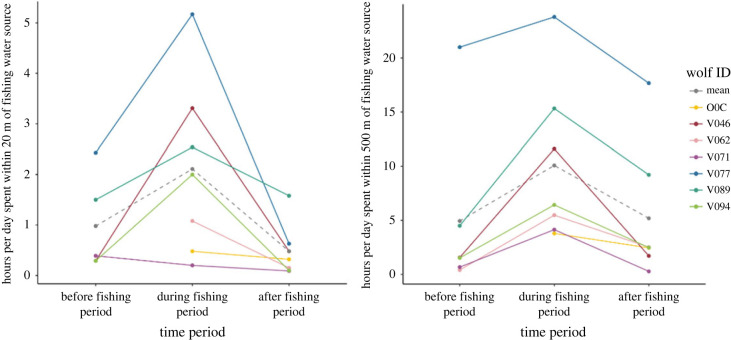
Hours per day wolves with 20 min fix interval collars (*n* = 7) spent within 20 m and 500 m of their fishing water source. We presumed that during each wolf's fishing period, hours spent within 20 m and 500 m of their fishing water source represent hours spent hunting fish, as well as hunting and handling fish respectively.

For wolves with 20 min fix interval collars (*n* = 7; table 2), we considered the number of GPS locations within 20 m and 500 m of a fishing water source as an index of the time wolves spent hunting or hunting *and* handling fish respectively during their fishing period. Wolves spent more hours per day on average within these buffers during their fishing period (2.11 ± 1.75 (s.d.) h within 20 m buffer and 10.8 ± 7.39 (s.d.) h within 500 m buffer) than the same length of time before (0.98 ± 0.96 (s.d.) h within 20 m buffer and 4.94 ± 8 (s.d.) hours within 500 m buffer) or after (0.48 ± 0.53 (s.d.) h within 20 m buffer and 5.19 ± 6.19 (s.d.) h within 500 m buffer) their fishing periods (figure 4). We did not conduct a statistical test on these differences because the sample size was small. All wolves except one (V071) spent more hours per day within 20 m of their fishing water source during their fishing period than before or after. All wolves, including V071, spent more hours per day within 500 m of their fishing water source during their fishing period than before or after. V077 spent the most hours per day within their 20 and 500 m buffers during their fishing period (5.17 and 23.8 h respectively). V062 did not spend any time within 20 m of their fishing water source before their fishing period, but did spend time within 500 m of their fishing water source before their fishing period (0.4 h per day).

### 3.1. Remote camera data

We recorded 59 separate remote camera events of wolves fishing in or near Irwin Creek in 2018 and 2019. We observed collared wolves in 37% (*n* = 22) of fishing events. In 63% (*n* = 37) of events, we observed uncollared wolves or unknown wolves (i.e. wolves we could not determine if they were collared or not).

#### 3.1.1. Fishing behaviours captured by remote video cameras

Of the 59 events recorded, we categorized 2% (*n* = 1) of the videos as *successful fishing attempts*, 3% (*n* = 2) as *failed fishing attempts*, and 2% (*n* = 1) as *unknown fishing attempts.* Five per cent (*n* = 3) depicted wolves *caching fish* on the stream bank, 2% depicted wolves *carrying fish* (*n* = 1), 34% (*n* = 20) depicted wolves *wading* in the creek and 53% (*n* = 31) depicted wolves *on the creek bank*.

#### 3.1.2. Description of fishing behaviours captured on remote cameras

Here, we provide a written description and summary of fishing behaviour observed on remote cameras. The observed hunting sequence consisted of wolves waiting on the creek bank or wading in shallow water less than 1 m deep, visually or auditorily (electronic supplementary material, video S3) detecting a fish, and then ambushing the fish by plunging their snout into the water.

**Figure 5.**
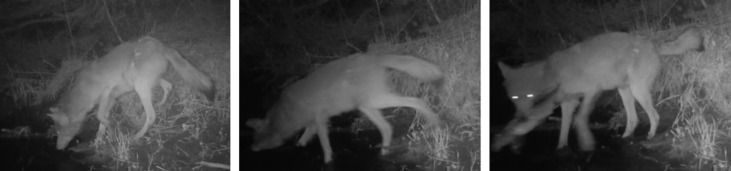
Images of an uncollared wolf ambushing a freshwater fish. Images are from a remote camera video recorded at Irwin Creek in northern Minnesota in May 2018.

One event captured a *successful fishing attempt* from an uncollared wolf (figure 5; electronic supplementary material, video S1). In the video, the wolf stood on the creek bank. The wolf then stepped into the creek, successfully caught a fish with its mouth, then walked onto the creek bank and out of frame.

In a video taken 3 min after the *successful fishing attempt*, the wolf returned to the bank until it appeared to hear a splash that is audible in the video, which caused the wolf to wade back into the water (electronic supplementary material, video S3). One minute later, the wolf returned to the creek bank and heard another splash. The wolf promptly waded back into the water again and lunged at a fish out of the camera frame.

Within the next 30 min following the end of this *successful fishing attempt*, the same remote camera recorded two separate video events of an uncollared wolf *caching* fish (electronic supplementary material, video S1). Three events in total, including the two mentioned above, captured uncollared wolves *caching* fish they caught. In all three events, the wolf exited the creek and dropped a single fish onto the bank. In two of these events, the fish was clearly alive and writhing until the wolf killed the fish by biting down on it before subsequently dropping it again. In two of the three *caching* events, the wolf returned to the creek after *caching* the fish on shore.

We recorded two *unsuccessful fishing attempts*, one of an uncollared wolf and one of a collared wolf (V034; electronic supplementary material, video S1). In the video of the uncollared wolf, the wolf stood on the edge of the creek with its front paws in the water actively looking into the creek. A fish swam by, and the wolf unsuccessfully attempted to capture it. In the event depicting the collared wolf's *unsuccessful fishing attempt,* the wolf chased a fish in a circle around a shallow section of Irwin Creek. After failing to capture the fish, the wolf exited the creek. Shortly thereafter, the wolf briefly returned to the creek before leaving again.

We recorded an *unknown fishing attempt* from an uncollared wolf in 2018 in which the wolf was on the creek bank with its front paws in the water and then lunged forward into the creek and out of the video frame (electronic supplementary material, video S1). A fish swam by just after the wolf left the frame.

One event in 2018 captured an uncollared wolf *carrying a fish* (electronic supplementary material, video S1). The event began with the wolf on the bank walking up from the creek with a fish in its mouth. The wolf then trotted out of the frame still carrying the fish in its mouth.

### 3.2. Camera collar data

**Figure 6.**
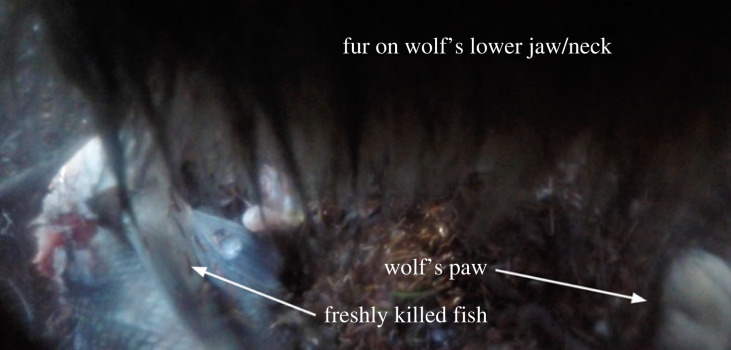
An image taken from camera collar footage of wolf V089 eating a freshly killed fish in May 2020.

We recorded six videos via camera collar of a wolf attempting to catch fish and 12 videos of the wolf eating or standing at a fish kill (figure 6; electronic supplementary material, video S2). These 18 videos of fishing behaviour constituted 42% of all camera collar videos associated with hunting and eating prey during the 43 d deployment period, and 2% of all videos recorded by the camera collar during this time.

## 4. Discussion

Our study describes in detail the behaviour of wolves hunting freshwater fishes in a boreal forest ecosystem using a diverse set of data and methods. This work provides further evidence that wolves are adept at quickly responding to alternate prey, despite the challenges with documenting these short-term responses in a cryptic carnivore. Additionally, although wolf ambush hunting behaviour has historically received less attention than cursorial hunting behaviour [41,42] our research suggests that both ambushing and fishing are not as rare a behaviour for wolves as previously thought [27], and may be widespread across similar ecosystems.

Wolves generally fished in shallow waterways (less than 1 m deep) probably allowing wolves to hunt without fish detecting their presence before an attack is launched, as well as limiting the ability of fish to escape. Deeper water probably reduces wolves' ability to detect and successfully capture fish. Our observations are similar to those of wolves hunting salmon in British Columbia where wolves were recorded fishing in water no more than 0.5 m deep [16].

Environmental conditions and landscape features can facilitate wolf fishing behaviour by reducing water levels and making fish more accessible. We observed several wolves in different years spending substantial time hunting fish in the shallow area just downstream of beaver dams, indicating dams create favourable fishing conditions for wolves. Beavers build dams that become physical barriers for fish travelling upstream, and often fish congregate below these dams as a result [43]. In addition, dams create shallow downstream waterways that make fish more vulnerable to predation [44,45]. Beaver colonies and associated dams are numerous in the GVE and across the boreal ecosystem [46]. Our observations indicate that beavers, by constructing dams, can create favourable conditions for catching fish and thus indirectly facilitate fishing behaviour by wolves.

In 2021, a major drought in Minnesota resulted in lower-than-average water levels across the state [47]. The drought conditions reduced many waterways to a small trickle by mid-summer and appeared to concentrate and trap fish in shallow pools or sections of rivers and creeks. The only wolf documented fishing outside of the April–May period (wolf O0C) occurred during this drought. Although only a single individual, we suspect that low water levels can facilitate fishing behaviour later in the summer [22,23], though future research should investigate the implications of seasonal water levels on wolf fishing behaviour.

Wolves clearly used ambush strategies to catch fish, adding to growing evidence that wolf predation is more flexible than the cursorial behaviours primarily used to hunt ungulates [41]. Indeed, the fishing behaviour we observed is similar to the ambush strategies wolves use to hunt salmon [16,48], and beavers [41]. Our video evidence suggests that auditory cues are particularly important for this ambush behaviour in the GVE, given the fact that wolves hunt fish at night when visual cues may be inhibited. Interestingly, on multiple occasions the sound of a surfacing fish caused wolves to quickly return to the creek in an attempt to catch fish (electronic supplementary material, video S3). A written account of how wolves hunt salmon indicates ‘Once a salmon splashed in the shallow stream and two of them [wolves] raced after it’ ([26], within [48, pp. 155]). The splash from a fish is probably an important cue for launching an attack and is possibly why wolves target shallower areas where prey are more easily detected.

Previous accounts of wolves fishing are largely limited to behavioural observations recorded during daylight hours [16], but the observations presented here suggest that wolves use auditory senses at night in a similar manner as black (*U. americanus*) and brown bears (*Ursus arctos*). Both bear species showed an increase in capture efficiency during twilight or darkness compared to daylight hours [49,50]. This is largely owing to the fact that salmon generate auditory cues such as splashing while they spawn that predators cue in on, and schools of salmon are less responsive to shoreline intrusion at night [40,50]. Consequently, black and brown bears shift from relying on visual cues during day to relying on auditory cues during night to locate and hunt salmon, a behavioural pattern we suggest wolves probably use when fishing too.

Wolves can probably recognize and respond to the short-term daily availability of fish by catching, killing and caching them on shorelines when available at night, and then consuming the fish when they are not available during the day as a way to maximize the number of fish caught (i.e. optimize resource acquisition rates). This behaviour follows the rapid-sequestering hypothesis, where an animal caches a temporarily abundant food source to maximize harvest rate while decreasing travel costs [51,52]. Wolves in British Columbia were observed carrying salmon into nearby forests or estuaries, but whether fish were immediately consumed was unknown [16]. Similar behaviour was observed in red foxes (*Vulpes vulpes*), where an individual left, hid or buried captured European carp (*Cyprinus carpio*) approximately 20–30 m from the shore of a reservoir [53]. We only observed three video events of wolves caching fish, so it is unclear whether the behaviour is common.

The spring spawning season aligns with a time of year when wolves have low ungulate hunting success [48,54]. For wolves, spring is between good adult deer hunting in the winter, when wolves have an advantage over their prey in deep or crusted snow, and the arrival of vulnerable and readily available fawns in early summer [54]. Throughout this period of decreased deer availability in spring, pups are born [54]. We found fish remains at den sites used by the Bowman Bay Pack in 2017 and the Half-Moon Pack in 2021 (D. R. Freund, T. D. Gable, and A. T. Homkes 2021, unpublished data), indicating that wolves may provision pups with fish. Additionally, four of the collared wolves fishing were breeding males that frequently returned to their dens right after fishing, presumably to provision the nursing female and/or pups. Seasonal fish runs within the GVE may therefore serve as a valuable pulsed resource to wolves when deer availability is low and packs have greater energetic demands because of newly born pups.

Once fish become available, wolves appear to spend the majority of their time hunting and killing fish [27]. This focus on catching fish probably leads to a corresponding decrease in predation of other primary prey species such as beavers and deer. For example, using methods described by [46], we determined that a young male wolf (V077) killed three beavers from 7–14 May before fishing from 19–29 May, during which time fish were the only prey remains we documented while searching clusters from this wolf. Fish are probably a more energetically profitable and safer prey to hunt and kill than beavers because they require less search time, less energy to acquire and consume, and have less potential to inflict injury [16,54]. Even beavers that are caught on land, where they were once assumed to be defenceless, can be difficult to kill [48,55]. To understand the proportion of diet that freshwater fishes make up during the spring spawning season, future studies can incorporate the use of scat or stable isotope analysis across multiple packs. Although this will not elucidate if wolves actively hunted versus scavenged fish, it will increase our understanding of the importance freshwater fish play in the annual diet of wolves and the degree of dietary plasticity in response to a pulsed resource.

Our observations indicate that fishing behaviour can develop individually or be learned from other pack members, providing insight into how fishing might arise and persist in wolf populations. We observed multiple generations of fishing wolves at the same creek within the Bowman Bay Pack from 2017 to 2019, suggesting that social learning may be responsible in part for perpetuating fishing behaviour. Offspring from breeding animals that fish may be more likely to fish when they are adults. However, the initial development of fishing may be an opportunistic response to available resources, whereby individual wolves can independently develop this behaviour given the right conditions. For example, in 2019, we studied V077's predation behaviour from early spring to autumn, during which time the wolf never fished. In 2020, the wolf became the breeding male of a newly formed pack and hunted and killed fish by himself. The only other pack-member, his mate, was collared and studied as well but she did not hunt or kill fish. Thus, the fishing behaviour by V077 appeared to develop independently of other individuals. Additionally, the 10 collared wolves we observed fishing were from five different social groups. This social variety suggests that wolf fishing behaviour arose across these individuals separately by opportunistic response to available resources.

Through the amalgamation of opportunistic data presented here, we provide a detailed description of a cryptic and brief behaviour, occurring on average less than 20 days of the year. Although we do not suspect that fish play a significant role in annual wolf diet owing to the brevity of the spawning season, understanding the extent of wolf ambushing behaviour and foraging flexibility can provide important life-history information and guide conservation and management efforts [18,27,56]. Grey wolves are recolonizing regions in North America and Eurasia for the first time in decades, and knowledge of their prey selection will aid in predicting future ecological impacts of wolves, as well as avoid surprises that may undermine conservation and restoration goals [14]. Additionally, the fishing behaviour of wolves appears sensitive to changes in water levels, and thus human-induced changes to local water levels via direct (e.g. human-built dams, removal of beaver dams) or indirect mechanisms (e.g. climate change) are likely to influence the frequency of wolf fishing behaviour. We expect that future research investigating wolf predation on freshwater fish populations will reveal this behaviour elsewhere across wolf range, and continue to elucidate wolves' ability to respond to ephemeral fluctuations in secondary prey.

## Ethics

The study followed Institutional Animal Care and Use Committee approvals by the U.S. National Park Service (MWR VOYA WINDELS WOLF) and the University of Minnesota (protocol ID: 1905-37051A).

## Data Availability

The code is provided at Dani Freund (2023): DaniFreund/ethology_of_wolves_freshwater_fishing: v1 (version v1), Zenodo, see: https://doi.org/10.5281/zenodo.7896841 [57]. Data includes sensitive wolf den site information and can be provided upon request. Supplementary material can be found in [58].

## References

[1] Bonin M, Dussault C, Taillon J, Pisapio J, Lecomte N, Côté SD. 2023 Diet flexibility of wolves and black bears in the range of migratory caribou. J. Mammal. **104**, 252-264. (10.1093/jmammal/gyad002)

[2] Doherty TS et al. 2019 Continental patterns in the diet of a top predator: Australia's dingo. Mammal Rev. **49**, 31-44. (10.1111/mam.12139)

[3] Popp JN, Hamr J, Larkin JL, Mallory FF. 2018 Black bear (*Ursus americanus*) and wolf (*Canis* spp.) summer diet composition and ungulate prey selectivity in Ontario, Canada. Mammal Res. **63**, 433-441. (10.1007/s13364-018-0368-y)

[4] Ward EJ, Levin PS, Lance MM, Jeffries SJ, Acevedo-Gutiérrez A. 2012 Integrating diet and movement data to identify hot spots of predation risk and areas of conservation concern for endangered species: identifying hot spots of predation risk. Conserv. Lett. **5**, 37-47. (10.1111/j.1755-263X.2011.00210.x)

[5] Ostfeld RS, Keesing F. 2000 Pulsed resources and community dynamics of consumers in terrestrial ecosystems. Trends Ecol. Evol. **15**, 232-237. (10.1016/S0169-5347(00)01862-0) 10802548

[6] Yang LH, Bastow JL, Spence KO, Wright AN. 2008 What can we learn from resource pulses. Ecology **89**, 621-634. (10.1890/07-0175.1) 18459327

[7] Bastille-Rousseau G, Rayl ND, Ellington EH, Schaefer JA, Peers MJL, Mumma MA, Mahoney SP, Murray DL. 2016 Temporal variation in habitat use, co-occurrence, and risk among generalist predators and a shared prey. Can. J. Zool. **94**, 191-198. (10.1139/cjz-2015-0127)

[8] Karandikar H, Serota MW, Sherman WC, Green JR, Verta G, Kremen C, Middleton AD. 2022 Dietary patterns of a versatile large carnivore, the puma (*Puma concolor*). Ecol. Evol. **12**, e9002. (10.1002/ece3.9002) 35784054 PMC9240727

[9] Wiebe N et al. 2009 Foraging behaviours and diets of wolves in the queen maud gulf bird sanctuary, Nunavut, Canada. Arctic **62**, 399-404.

[10] Mahmood T. 2021 Diet composition of grey wolf (*Canis lupus*) varies seasonally in Deosai National Park, Gilgit-Baltistan, Pakistan. Pak. J. Zool. **54**, pp. 1-9. (10.17582/journal.pjz/20210406040405)

[11] Gable TD, Windels SK, Bruggink JG, Barber-Meyer SM. 2018 Weekly summer diet of gray wolves (*Canis lupus*) in northeastern Minnesota. Am. Midl. Nat. **179**, 15-27. (10.1674/0003-0031-179.1.15)

[12] Homkes AT, Gable TD, Windels SK, Bump JK. 2020 Berry important? Wolf provisions pups with berries in Northern Minnesota. Wildl. Soc. Bull. **44**, 221-223. (10.1002/wsb.1065)

[13] Darimont CT, Reimchen TE. 2002 Intra-hair stable isotope analysis implies seasonal shift to salmon in gray wolf diet. Can. J. Zool. **80**, 1638-1642. (10.1139/z02-149)

[14] Newsome TM et al. 2016 Food habits of the world's grey wolves. Mammal Rev. **46**, 255-269. (10.1111/mam.12067)

[15] Adams LG, Farley SD, Stricker CA, Demma DJ, Roffler GH, Miller DC, Rye RO. 2010 Are inland wolf–ungulate systems influenced by marine subsidies of Pacific salmon? Ecol. Appl. **20**, 251-262. (10.1890/08-1437.1) 20349845

[16] Darimont CT, Reimchen TE, Paquet PC. 2003 Foraging behaviour by gray wolves on salmon streams in coastal British Columbia. Can. J. Zool. **81**, 349-353. (10.1139/z02-246)

[17] Darimont CT, Price MHH, Winchester NN, Gordon-Walker J, Paquet PC. 2004 Predators in natural fragments: foraging ecology of wolves in British Columbia's central and north coast archipelago. J. Biogeogr. **31**, 1867-1877. (10.1111/j.1365-2699.2004.01141.x)

[18] Darimont CT, Paquet PC, Reimchen TE. 2008 Spawning salmon disrupt trophic coupling between wolves and ungulate prey in coastal British Columbia. BMC Ecol. **8**, 14. (10.1186/1472-6785-8-14) 18764930 PMC2542989

[19] Stanek AE, Wolf N, Hilderbrand GV, Mangipane B, Causey D, Welker JM. 2017 Seasonal foraging strategies of Alaskan gray wolves (*Canis lupus*) in an ecosystem subsidized by Pacific salmon (*Oncorhynchus* spp.). Can. J. Zool. **95**, 555-563. (10.1139/cjz-2016-0203)

[20] Szepanski MM, Ben-David M, Van Ballenberghe V. 1999 Assessment of anadromous salmon resources in the diet of the Alexander Archipelago wolf using stable isotope analysis. Oecologia **120**, 327-335. (10.1007/s004420050866) 28308010

[21] Watts DE, Newsome SD. 2016 Exploitation of marine resources by wolves in southwestern Alaska. J. Mammal. **98**, 66-76. (10.1093/jmammal/gyw153)

[22] Kuyt E. 1969 Feeding ecology of wolves on barren-ground caribou range in the northwest territories. Masters thesis, University of Saskatchewan, Saskatoon, Saskatchewan, Canada.

[23] Kuyt E. 1972 Food habits of wolves on barren-ground caribou range. Canadian Wildlife Service Report Series - Number 21. Canadian Wildlife Service, Ottawa, Ontario, Canada, pp. 1-36.

[24] Hill EL. 1979 The ecology of the timber wolf (*Canis lupus* Linn.) in southern Manitoba - wilderness, recreational and agricultural aspects. Masters thesis, University of Manitoba, Winnepeg, Manitoba, Canada.

[25] Bromley RG. 1973 Fishing behavior of a wolf on the Taltson River, Northwest Territories. Can. Field-Nat. **87**, 301-303.

[26] Young SP, Goldman EA. 1944 Wolves of North America part 2. Washington, DC: American Wildlife Institute. See http://hdl.handle.net/1969.3/25114.

[27] Gable TD, Windels SK, Homkes AT. 2018 Do wolves hunt freshwater fish in spring as a food source? Mamm. Biol. **91**, 30-33. (10.1016/j.mambio.2018.03.007)

[28] Kallemeyn LW, Holmberg KL, Perry JA, Odde BY. 2003 Aquatic synthesis for Voyageurs National Park, information and technology report, USGS/BDR/ITR-2003-001, pp. 1-81.

[29] Gable TD, Windels SK, Bruggink JG, Homkes AT. 2016 Where and how wolves (*Canis lupus*) kill beavers (*Castor canadensis*). PLoS ONE **11**, e0165537. (10.1371/journal.pone.0165537) 27992441 PMC5167233

[30] Gogan PJP, Route WT, Olexa EM, Thomas N, Kuehn D, Prodruzny KM. 2004 Gray wolves in and adjacent to Voyageurs National Park, Minnesota: research and synthesis 1987–1991. Technical report NPS/MWR/NRTR/2004-01, pp. 1-68.

[31] Gable T, Homkes A, Bump J. 2022 In press. 2021–2022 Greater Voyageurs Ecosystem wolf pack and population size report. 1–34.

[32] Gable TD, Windels SK, Bruggink JG. 2017 The problems with pooling poop: confronting sampling method biases in wolf (*Canis lupus*) diet studies. Can. J. Zool. **95**, 843-851. (10.1139/cjz-2016-0308)

[33] Gable TD, Windels SK, Olson BT. 2017 Estimates of white-tailed deer density in Voyageurs National Park: 1989–2016. Natural Resource Report NPS/VOYA/NRR—2017/1427. National Park Service, Fort Collins, Colorado.

[34] Gable TD, Windels SK. 2018 Kill rates and predation rates of wolves on beavers. J. Wildl. Manag. **82**, 466-472. (10.1002/jwmg.21387)

[35] Windels SK, Olson BT. 2015 Voyageurs National Park moose population survey report. Natural resource data series NPS/VOYA/NRDS-2015/971, pp. 1–7.

[36] Chenaux-Ibrahim Y. 2015 Seasonal diet composition of gray wolves (*Canis lupus*) in northeastern Minnesota determined by scat analysis. Masters thesis, University of Minnesota, Minneapolis, MN, USA.

[37] Akbaba B, Ayaş Z. 2012 Camera trap study on inventory and daily activity patterns of large mammals in a mixed forest in north-western Turkey. Mammalia **76**, 43-48. (10.1515/mamm.2011.102)

[38] Iannarilli F, Arnold TW, Erb J, Fieberg JR. 2019 Using lorelograms to measure and model correlation in binary data: applications to ecological studies. Methods Ecol. Evol. **10**, 2153-2162. (10.1111/2041-210X.13308)

[39] Tallian A et al. 2022 Of wolves and bears: seasonal drivers of interference and exploitation competition between apex predators. Ecol. Monogr. **92**, e1498. (10.1002/ecm.1498)

[40] Reimchen TE. 1998 Nocturnal foraging behavior of black bears, *Ursus americanus*, on Moresby Island, British Columbia. Can. Field-Nat. **112**, 446-450.

[41] Gable TD, Homkes AT, Johnson-Bice SM, Windels SK, Bump JK. 2021 Wolves choose ambushing locations to counter and capitalize on the sensory abilities of their prey. Behav. Ecol. **32**, 339-348. (10.1093/beheco/araa147)

[42] Rossoni S, Niven JE. 2020 Prey speed influences the speed and structure of the raptorial strike of a ‘sit-and-wait’ predator. Biol. Lett. **16**, 20200098. (10.1098/rsbl.2020.0098) 32396788 PMC7280040

[43] Johnson-Bice SM, Renik KM, Windels SK, Hafs AW. 2018 A review of beaver-salmonid relationships and history of management actions in the Western Great Lakes (USA) Region. North Am. J. Fish. Manag. **38**, 1203-1225. (10.1002/nafm.10223)

[44] Gurnell AM. 1998 The hydrogeomorphological effects of beaver dam-building activity. Prog. Phys. Geogr. Earth Environ. **22**, 167-189. (10.1177/030913339802200202)

[45] Smith JM, Mather ME. 2013 Beaver dams maintain fish biodiversity by increasing habitat heterogeneity throughout a low-gradient stream network. Freshw. Biol. **58**, 1523-1538. (10.1111/fwb.12153)

[46] Gable TD, Johnson-Bice SM, Homkes AT, Windels SK, Bump JK. 2020 Outsized effect of predation: wolves alter wetland creation and recolonization by killing ecosystem engineers. Sci. Adv. **6**, eabc5439. (10.1126/sciadv.abc5439) 33188026 PMC7673763

[47] Davenport MA, Kreiter A, Brauman KA, Keeler B, Arbuckle J, Sharma V, Pradhananga A, Noe R. 2022 An experimental model of drought risk and future irrigation behaviors among central Minnesota farmers. Clim. Change **171**, 8. (10.1007/s10584-022-03320-3)

[48] Mech LD, Smith DW, MacNulty DR. 2015 Wolves on the hunt. Chicago, USA: University of Chicago Press.

[49] Klinka DR, Reimchen TE. 2002 Nocturnal and diurnal foraging behaviour of brown bears (*Ursus arctos*) on a salmon stream in coastal British Columbia. Can. J. Zool. **80**, 1317-1322. (10.1139/z02-123)

[50] Klinka DR, Reimchen TE. 2009 Darkness, twilight, and daylight foraging success of bears (*Ursus americanus*) on salmon in coastal British Columbia. J. Mammal. **90**, 144-149. (10.1644/07-MAMM-A-200.1)

[51] Careau V, Giroux J-F, Berteaux D. 2007 Cache and carry: hoarding behavior of Arctic fox. Behav. Ecol. Sociobiol. **62**, 87-96.

[52] Jenkins SH, Peters RA. 1992 Spatial patterns of food storage by Merriam's kangaroo rats. Behav. Ecol. **3**, 60-65. (10.1093/beheco/3.1.60)

[53] Tobajas J, Díaz-Ruiz F. 2022 Fishing behavior in the red fox: opportunistic-caching behavior or surplus killing? Ecology **103**, e3814. (10.1002/ecy.3814) 35841191 PMC10078576

[54] Mech LD. 2012 The wolf: the ecology and behavior of an endangered species. Minneapolis, MN: University of Minnesota Press.

[55] Gable TD, Stanger T, Windels SK, Bump JK. 2018 Do wolves ambush beavers? Video evidence for higher-order hunting strategies. Ecosphere **9**, e02159. (10.1002/ecs2.2159)

[56] Yirga G, De Iongh HH, Leirs H, Gebrihiwot K, Deckers J, Bauer H. 2012 Adaptability of large carnivores to changing anthropogenic food sources: diet change of spotted hyena (*Crocuta crocuta*) during Christian fasting period in northern Ethiopia: adaptability of large carnivores to changing anthropogenic food sources. J. Anim. Ecol. **81**, 1052-1055. (10.1111/j.1365-2656.2012.01977.x) 22486435

[57] Freund DR, Gable TD, Johnson-Bice SM, Homkes AT, Windels SK, Bump JK. 2023 The ethology of wolves foraging on freshwater fish in a boreal ecosystem. Zenodo. (10.5281/zenodo.7896840) PMC10206451 37234502

[58] Freund DR, Gable TD, Johnson-Bice SM, Homkes AT, Windels SK, Bump JK. 2023 The ethology of wolves foraging on freshwater fish in a boreal ecosystem. Figshare. (10.6084/m9.figshare.c.6660391) PMC10206451 37234502

